# A network meta-analysis on the efficacy of targeted agents in combination with chemotherapy for treatment of advanced/metastatic triple-negative breast cancer

**DOI:** 10.18632/oncotarget.19102

**Published:** 2017-07-08

**Authors:** Long Ge, Yan Tang, Qiu-Ning Zhang, Jin-Hui Tian, Xiao-Hu Wang, Dawid Pieper, Bei Pan, Lun Li, Juan Ling, Zhi-Tong Bing, Ke-Hu Yang

**Affiliations:** ^1^ First Clinical Medical College of Lanzhou University, Lanzhou 730000, P.R. China; ^2^ Evidence-Based Medicine Center of Lanzhou University, Lanzhou 730000, P.R. China; ^3^ Key Laboratory of Evidence-Based Medicine and Knowledge Translation of Gansu Province, Lanzhou 730000, P.R. China; ^4^ Second People's Hospital of Lanzhou City, Lanzhou 730046, P.R. China; ^5^ Gansu Provincial Academic Institute for Medical Research, Gansu Provincial Cancer Hospital, Lanzhou 730050, P.R. China; ^6^ Institute for Research in Operative Medicine, Faculty of Health, School of Medicine, Witten/Herdecke University, 51109, Cologne, Germany; ^7^ School of Public Health of Lanzhou University, Lanzhou 730000, P.R. China; ^8^ Department of Breast-Thyroid Surgery, The Second Xiangya Hospital of Central South University, Changsha 410000, P.R. China; ^9^ Institute of Modern Physics of Chinese Academy of Sciences, Lanzhou 730000, P.R. China

**Keywords:** targeted agents, chemotherapy, triple-negative breast cancer, network meta-analysis, randomized controlled trials

## Abstract

**Objective:**

Our network meta-analysis aimed to determine the assistant efficacy of targeted therapy in combined with chemotherapy for advanced/metastatic triple-negative breast cancer (TNBC).

**Results:**

A total of 15 randomized controlled trials (RCTs), involving 2,410 patients, met our inclusion criteria. Eight targeted agents involving 11 treatment arms were included. The methodological quality of included RCTs was acceptable. The results of direct comparisons showed that progression-free survival (PFS) was significantly longer with bevacizumab+chemotherapy when compared to chemotherapy alone (hazard ratio [HR] = 0.62, 95% credible intervals [CrI]: 0.41–0.87). However, there were no statistically significant differences for all other direct comparison groups. The results of indirect comparison of different targeted agents revealed no significant differences regarding all outcomes of interest. According to ranking probabilities, all outcomes favored bevacizumab+chemotherapy and veliparib+chemotherapy. Bayesian and Frequentist network meta-analysis showed similar results, and the probability of bias of small-study effects was small.

**Materials and Methods:**

A comprehensive literature search in PubMed, EMBASE, the Cochrane Central Register of Controlled Trials (CENTRAL), Web of Science (via ISI Web of Knowledge), BIOSIS Previews (via ISI Web of Knowledge), and Chemical Abstracts (CA) was conducted to identify RCTs involving targeted agents in the treatment of advanced/metastatic TNBC. Two reviewers independently extracted related data and assessed the risk of bias of included studies. Bayesian network meta-analysis was conducted using R-3.3.2 software.

**Conclusions:**

Limited evidence showed that targeted agents combined with chemotherapy for advanced/metastatic TNBC were slightly effective. Further investigation of targeted therapies for TNBC is required to improve patient outcomes. The registration number was CRD42014014299.

## INTRODUCTION

Breast cancer (BC) is by far the most frequent cancer among women, with an estimated 232,670 new cancer cases diagnosed and 40,000 deaths in the United States in 2014 [1]. Triple-negative breast cancer (TNBC), which is characterized by the lack of estrogen/progesterone-receptor (ER/PR) and human epidermal growth factor receptor-2 (HER-2), accounts for 15 to 20% of all BC cases [2]. Because of the absence of specific treatment guidelines for TNBC [3], many clinicians consider TNBC as the most difficult type of BC to treat, and some patients think it is a death sentence [4]. Some studies have also demonstrated that the prognosis of TNBC is poor [5, 6].

Current cytotoxic drug chemotherapy is the mainstay of TNBC treatment despite the absence of a specific therapeutic target [7]. A recent meta-analysis also showed that the odds of pathologic complete response (pCR) to neoadjuvant chemotherapy were highest for the triple-negative in all breast cancer subtypes [8]. However, a standard chemotherapy regimen for adjuvant treatment of TNBC is yet to be established, and a high risk for recurrence and disease progression was found after chemotherapy. Therefore, there remains an urgent need to develop more therapeutic strategies, especially targeted therapies for TNBC [2, 7, 9].

Recently, two pairwise meta-analyses were performed to compare the efficacy of targeted therapy to conventional chemotherapy in patients with metastatic TNBC. The results showed that targeted therapy combined with chemotherapy was superior for progression-free survival (PFS) when compared to chemotherapy alone [10, 11]. However, there were many limitations for these studies, such as the lack of knowledge of the best molecular-targeted therapies and the real impact of using targeted therapy combined with chemotherapy in overall survival (OS). Obviously, it was difficult for randomized controlled trials (RCTs) and pairwise meta-analysis to integrate information on the relative efficacy of all available tested regimens [12].

Network meta-analysis has become increasingly popular to evaluate healthcare interventions, which estimate the relative effectiveness among all interventions and rank ordering of the interventions even if some head to head comparisons are lacking [13]. RCTs of some targeted agents for TNBC are currently available. Here, we have systematically conducted a Bayesian network meta-analysis to compare the PFS, OS, and overall response rate (ORR) of different targeted agents in combination with chemotherapy for treating TNBC and to rank the targeted agents.

## RESULTS

### Search results

**Figure 1:**
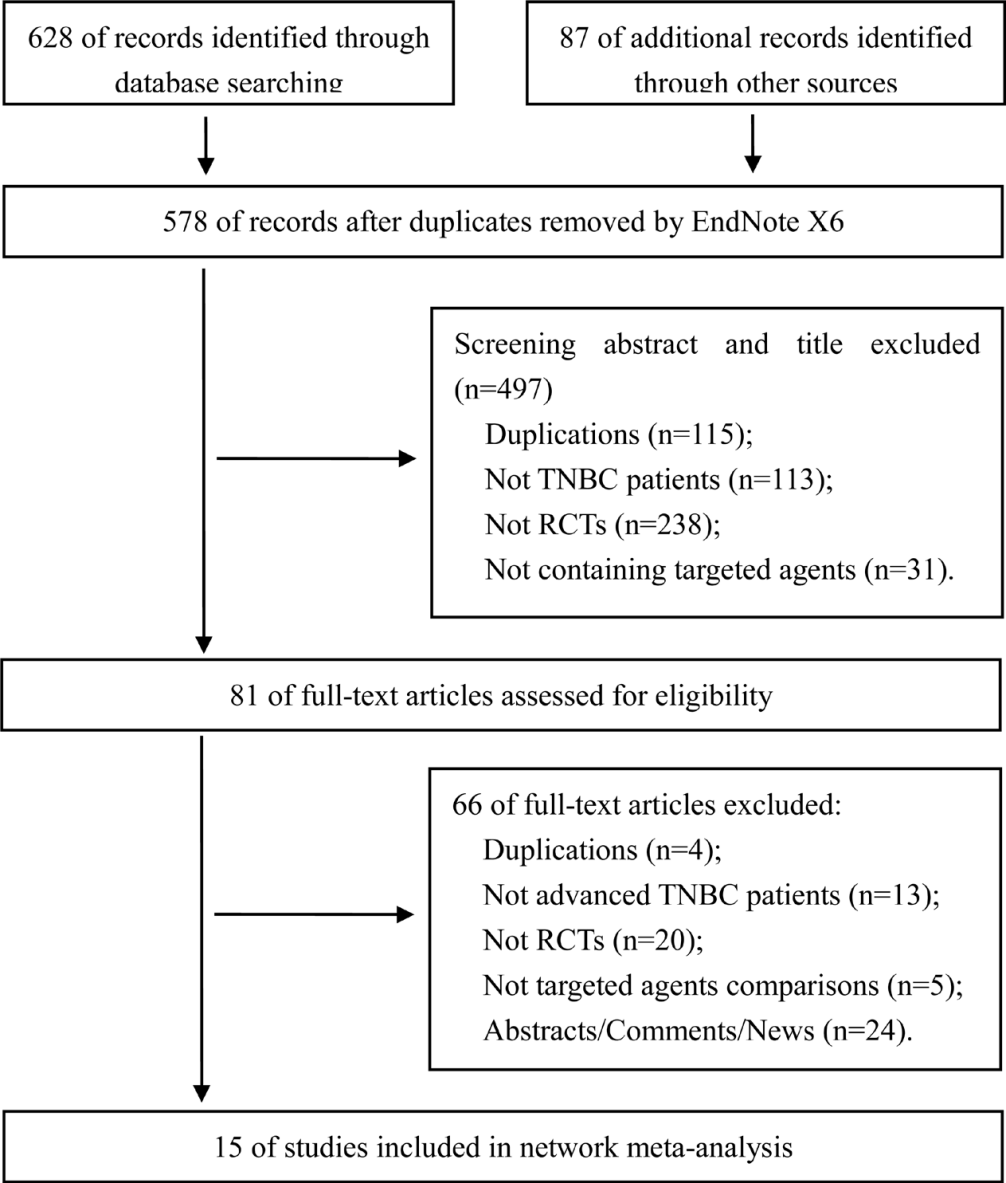
Search results and selection details

A total of 628 records were searched from electronic databases, and 5 systematic reviews [2, 10, 11, 14, 15], including 87 references, were tracked. Finally, 15 RCTs [16–30] involving 2,410 patients were included. The search results and selection details are shown in Figure 1.

### Characteristics of included studies

All included studies were multicenter studies. Fourteen RCTs reported the median PFS, and 8 RCTs reported the median OS. Fifteen RCTs, including 11 different treatment regimens, were assessed: bevacizumab+chemotherapy, iniparib+chemotherapy, lapatinib+chemotherapy, sunitinib, cetuximab+ chemotherapy, cetuximab, sorafenib+chemotherapy, sunitinib+chemotherapy, tigatuzumab chemotherapy, veliparib+chemotherapy, and chemotherapy alone. The details of included RCTs are presented in Table 1, and the details of chemotherapy regimens of included RCTs in Supplementary Table 1.

**Table 1:** Characteristics of included studies

Study	Arm	Sample	Median age	Median PFS (months)	Median OS (months)	Trial stage	Line of Treatment
Brufsky A 2011	bevacizumab+chemotherapy	112	55 (28–86)	6	17.9	III	2
	chemotherapy	47	49 (33–79)	2.7	12.6		
O’Shaughnessy J 2011	iniparib+chemotherapy	61	56 (34–76)	5.9 (4.5–7.2)	12.3 (9.8–21.5)	II	2
	chemotherapy	62	53 (26–80)	3.6 (2.6–5.2)	7.7 (6.5–13.3)		
Finn RS 2009	lapatinib+chemotherapy	71	NR	4.6 (3.9–5.3)	NR	III	1
	chemotherapy	60		4.8 (4.3–5.3)			
Curigliano G 2013	sunitinib	113	52 (32–81)	1.7 (1.5–2.6)	9.4 (5.8–11.2)	II	NR
	chemotherapy	104	52 (31–81)	2.5 (1.4–2.9)	10.5 (8.5–13.8)		
Trédan O 2014	cetuximab+chemotherapy	39	50 (31–79)	4.1 (2.7–6.1)		II	1
	chemotherapy	40	53 (29–75)	4.1 (3.0–4.9)			
Carey LA 2012	cetuximab	31	49 (33–71)	1.4 (1.1–1.8)	7.5 (5.0–11.6)	II	1,2,3
	cetuximab+chemotherapy	71	52 (28–83)	2.1 (1.8–5.5)	10.4 (7.7–13.1)		
Baselga J 2013	cetuximab+chemotherapy	115	53 ± 12.5	3.7 (2.8–4.3)	12.9 (9.6–15.6)	II	1,2
	chemotherapy	58	52 ± 10.7	1.5 (1.4–2.8)	9.4 (6.7–14.2)		
Baselga J 2012	sorafenib+chemotherapy	20	NR	4.3	17.5	IIB	1,2
	chemotherapy	33		2.5	16.1		
Bergh J 2012	sunitinib+chemotherapy	58	NR	NR	NR	III	1
	chemotherapy	69					
Pivot X 2011	bevacizumab+chemotherapy	113	NR	8.1	NR	III	1
	chemotherapy			6			
Miller K 2007	bevacizumab+chemotherapy	232	NR	10.6	NR	III	1
	chemotherapy			5.3			
Robert NJ 2011	bevacizumab+chemotherapy	87	NR	6.1	NR	III	1
	chemotherapy	50		4.2			
	bevacizumab+chemotherapy	96		6.5			
	chemotherapy	46		6.2			
O’Shaughnessy J 2014	iniparib+chemotherapy	261	53	5.1 (4.2–5.8)	12.2 (10.6–13.7)	III	1,2
	chemotherapy	258	54	4.1 (3.1–4.6)	11.1 (9.2–12.3)		
Forero-Torres A 2015	tigatuzumab+chemotherapy	42	51 (32–72)	2.8 (1.9–3.6)	NR	II	NR
	chemotherapy	22	51 (34–75)	3.7 (2.3–5.7)			
Kummar S 2016	veliparib+chemotherapy	21	54 (34–77)	2.1	NR	II	NR
	chemotherapy	18		1.9			

**Figure 2:**
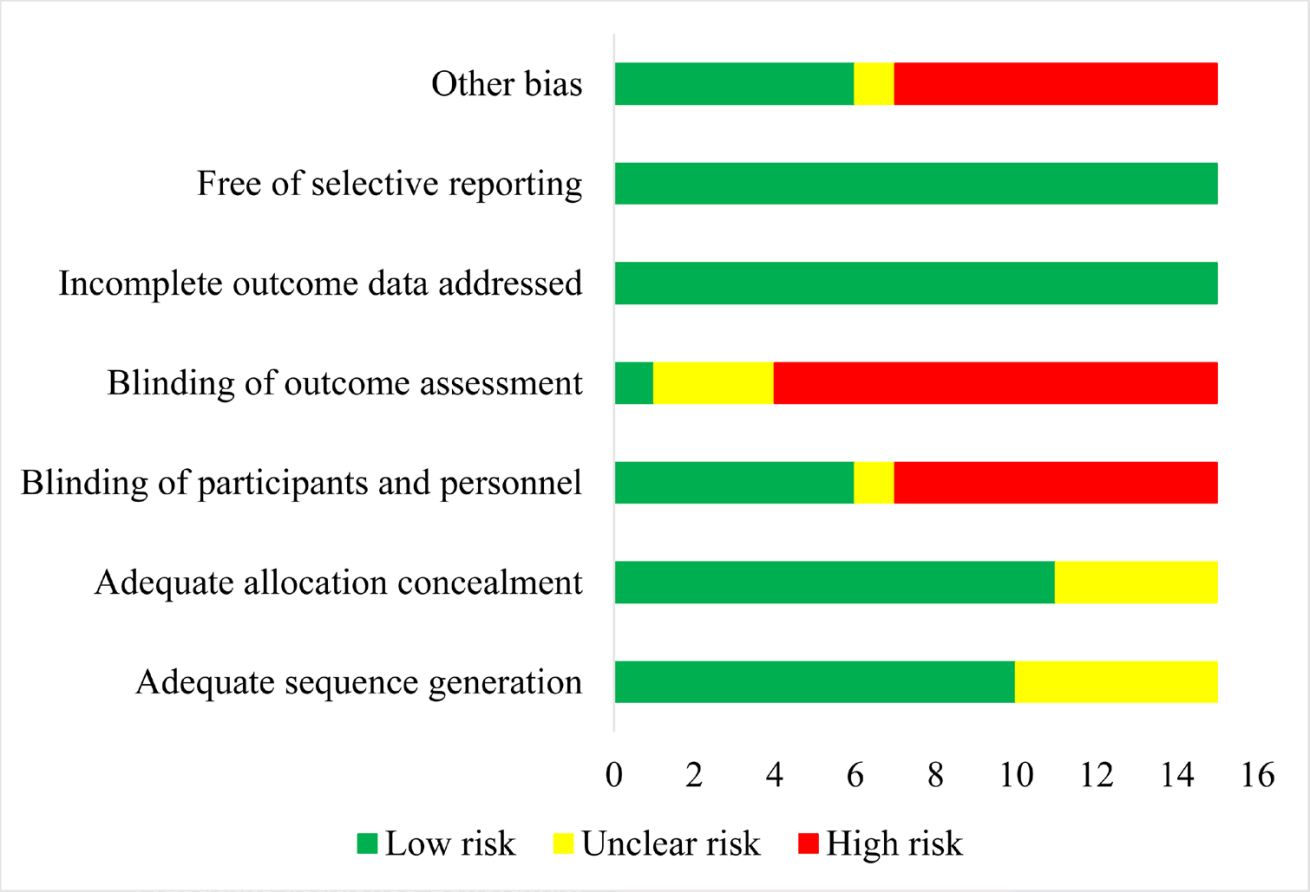
Results of risk of bias assessment

The results of assessment of risk of bias showed that most RCTs (66.7%) mentioned the methods of adequate sequence generation. Six RCTs had double-blind designs. Eight RCTs had open-label designs (Figure 2, Supplementary Table 2).

### Network meta-analyses

### Analysis of heterogeneity and inconsistency

For PFS, four studies [16, 25–27] compared bevacizumab+chemotherapy to chemotherapy alone, and significant heterogeneity was detected (I^2^ = 90.3%). Two studies [20–22] compared cetuximab+chemotherapy to chemotherapy alone, and significant heterogeneity was detected (I^2^ = 64.4%). Two studies [17, 28] compared iniparib+chemotherapy to chemotherapy alone, and found no evidence of heterogeneity (I^2^ = 0.0%). For OS and ORR, there was no evidence of heterogeneity in terms of all comparison groups. A random effect model Bayesian network meta-analysis was performed. The inconsistency between direct and indirect comparisons was not assessed because there were no loops connecting three arms.

### PFS

**Figure 3:**
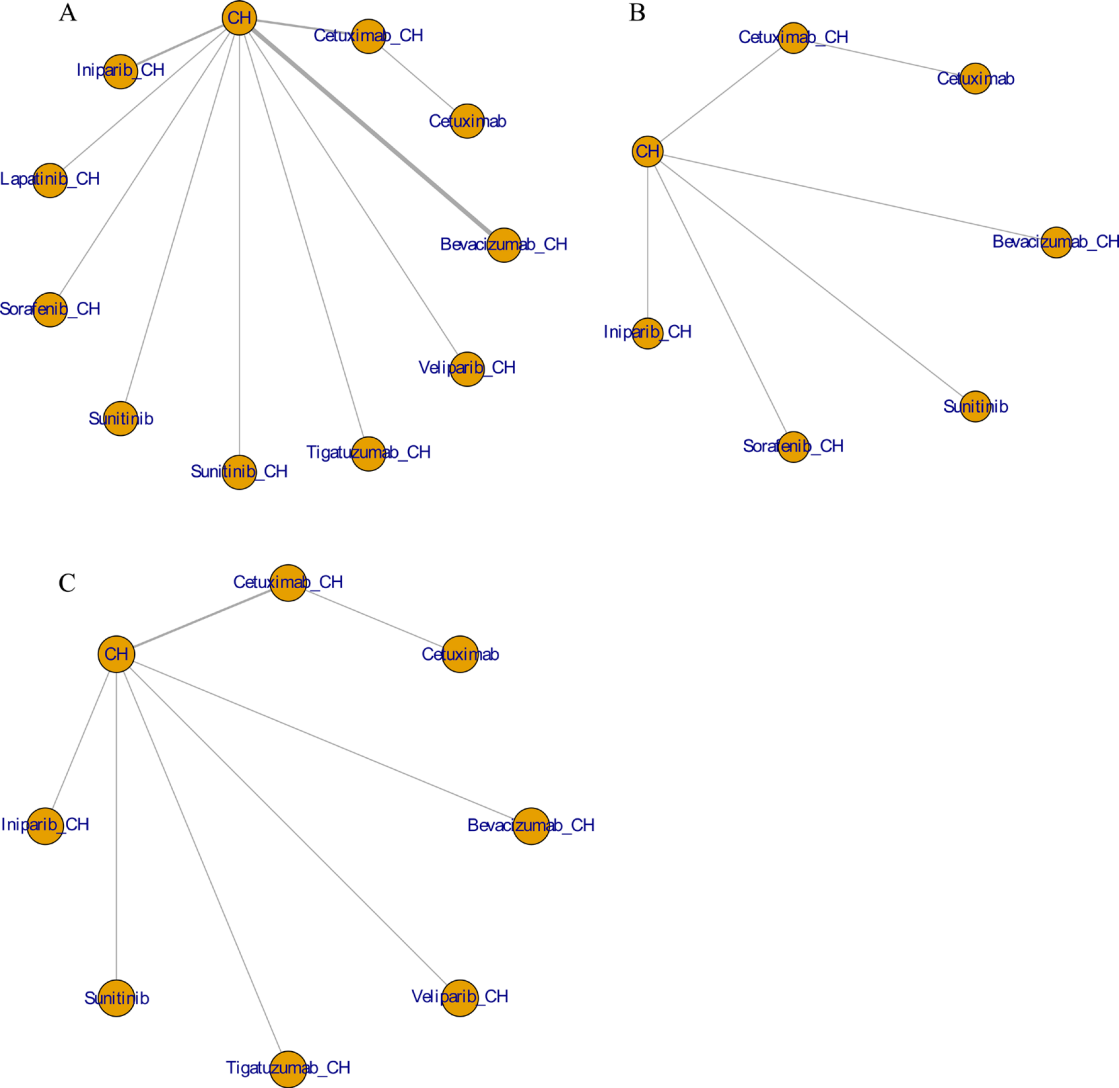
Network plots for PFS (**A**), OS (**B**), and ORR (**C**).

**Figure 4:**
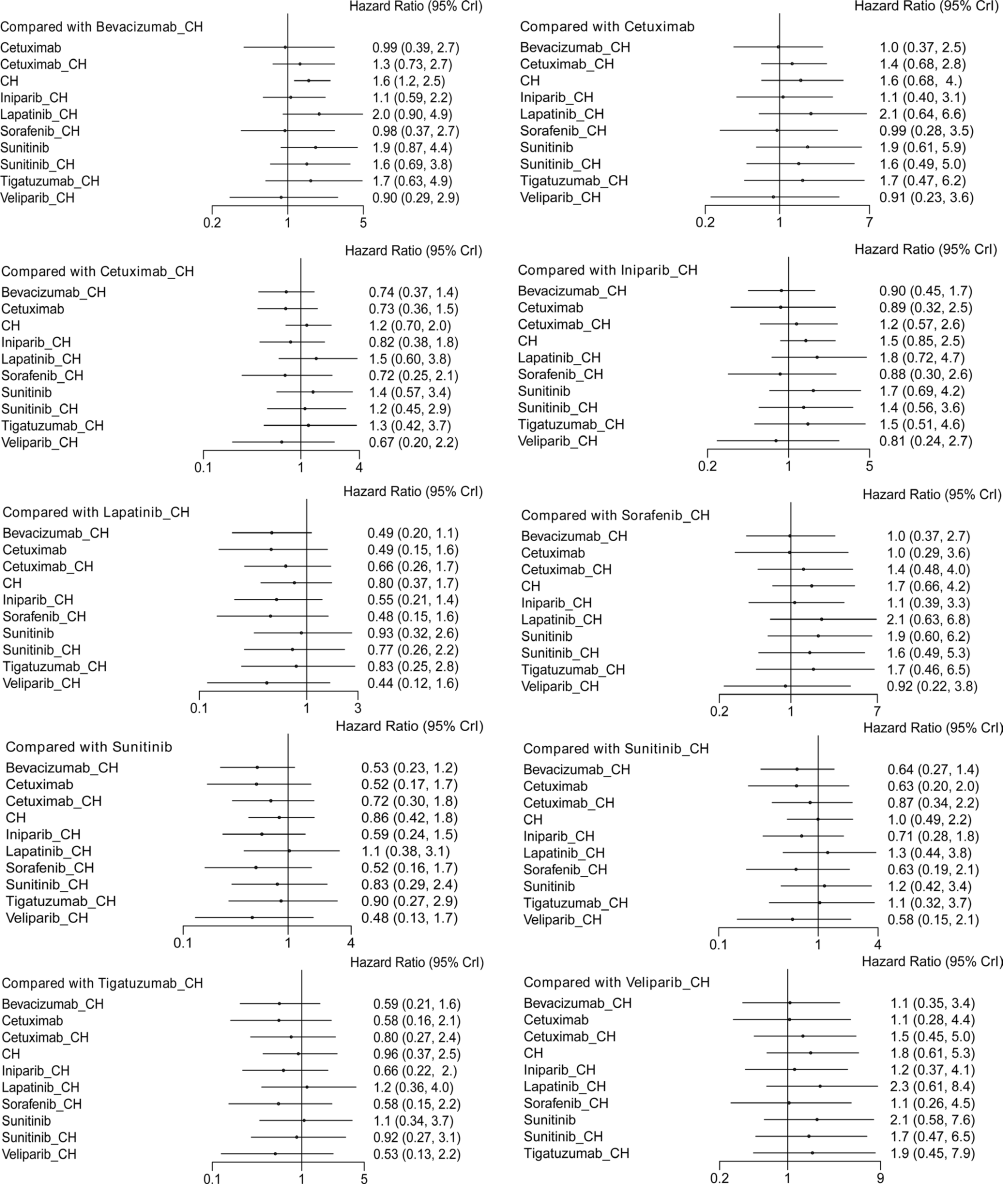
Results of network meta-analysis for PFS

**Figure 5:**
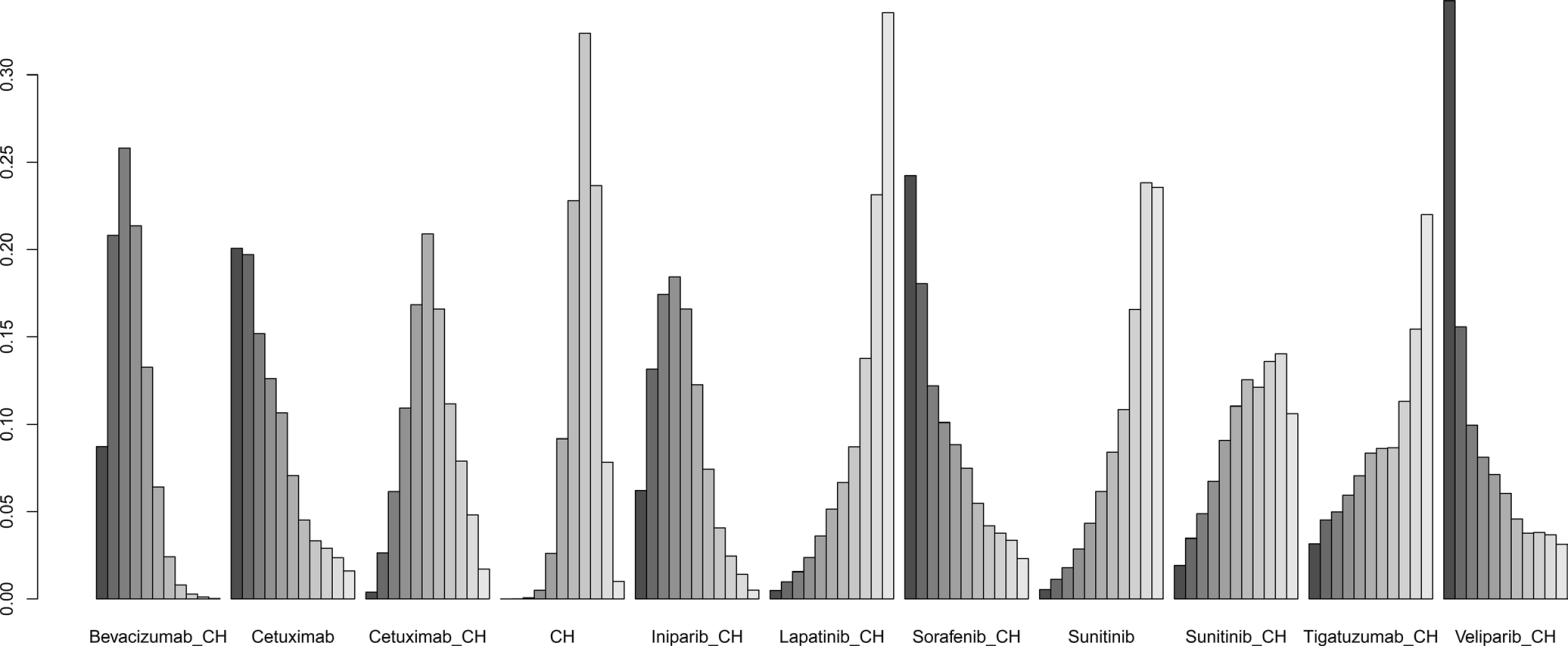
Results of treatment rank for PFS

Fifteen studies [16–30] (2,410 patients), involving 11 treatment arms, reported on PFS, (Figure 3A). The results of network meta-analysis showed that PFS was significantly longer with bevacizumab+chemotherapy when compared with chemotherapy alone (hazard ratio [HR] = 0.62, 95% credible interval [CrI]: 0.41–0.87). However, there were no statistically significant differences for all other comparison groups (Figure 4). According to the results of treatment rank probabilities, veliparib+chemotherapy had the highest probability of being the best treatment arm, followed by sorafenib+chemotherapy, cetuximab, and bevacizumab+chemotherapy (Figure 5).

### OS

**Figure 6:**
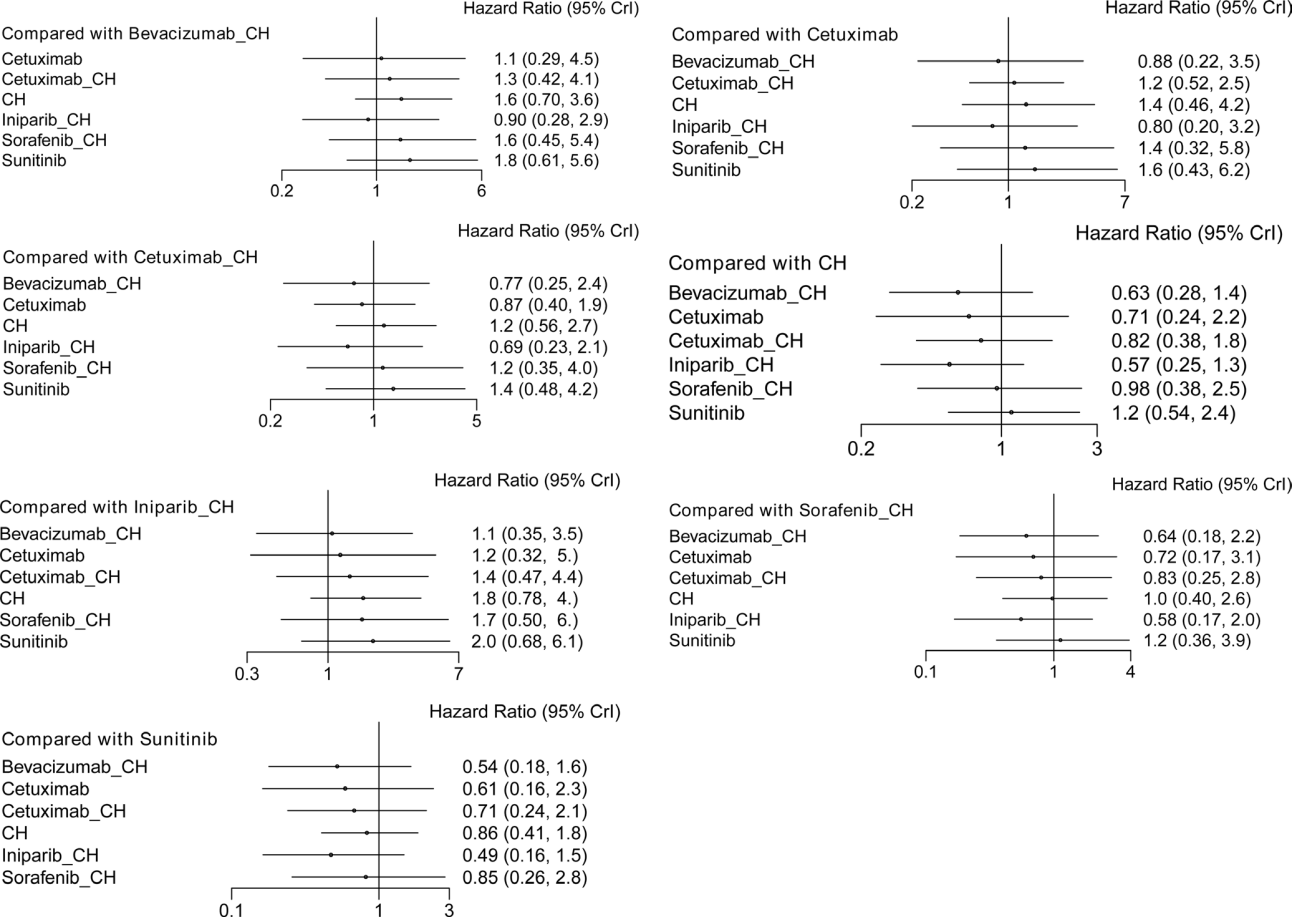
Results of network meta-analysis for OS

**Figure 7:**
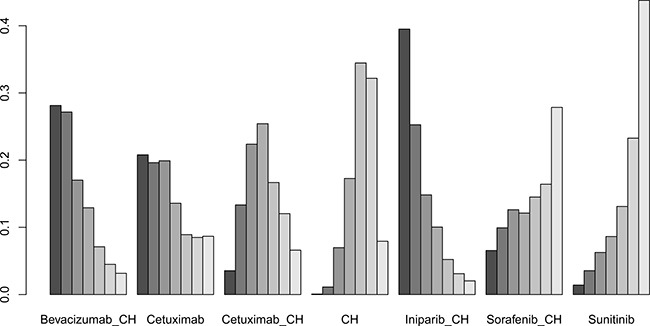
Results of treatment rank for OS

Six studies [16, 17, 19, 21–23] (827 patients), involving seven treatment arms, reported on OS, (Figure 3B). There were no statistically significant differences for all comparison groups in the improvement of OS (Figure 6). According to the results of treatment rank probabilities, iniparib+chemotherapy had the highest probability of being the best treatment arm, followed by bevacizumab+chemotherapy and cetuximab (Figure 7).

### ORR

**Figure 8:**
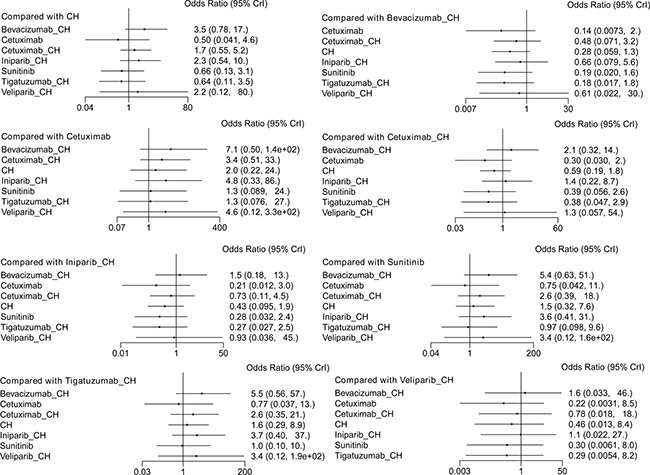
Results of network meta-analysis for ORR

**Figure 9:**
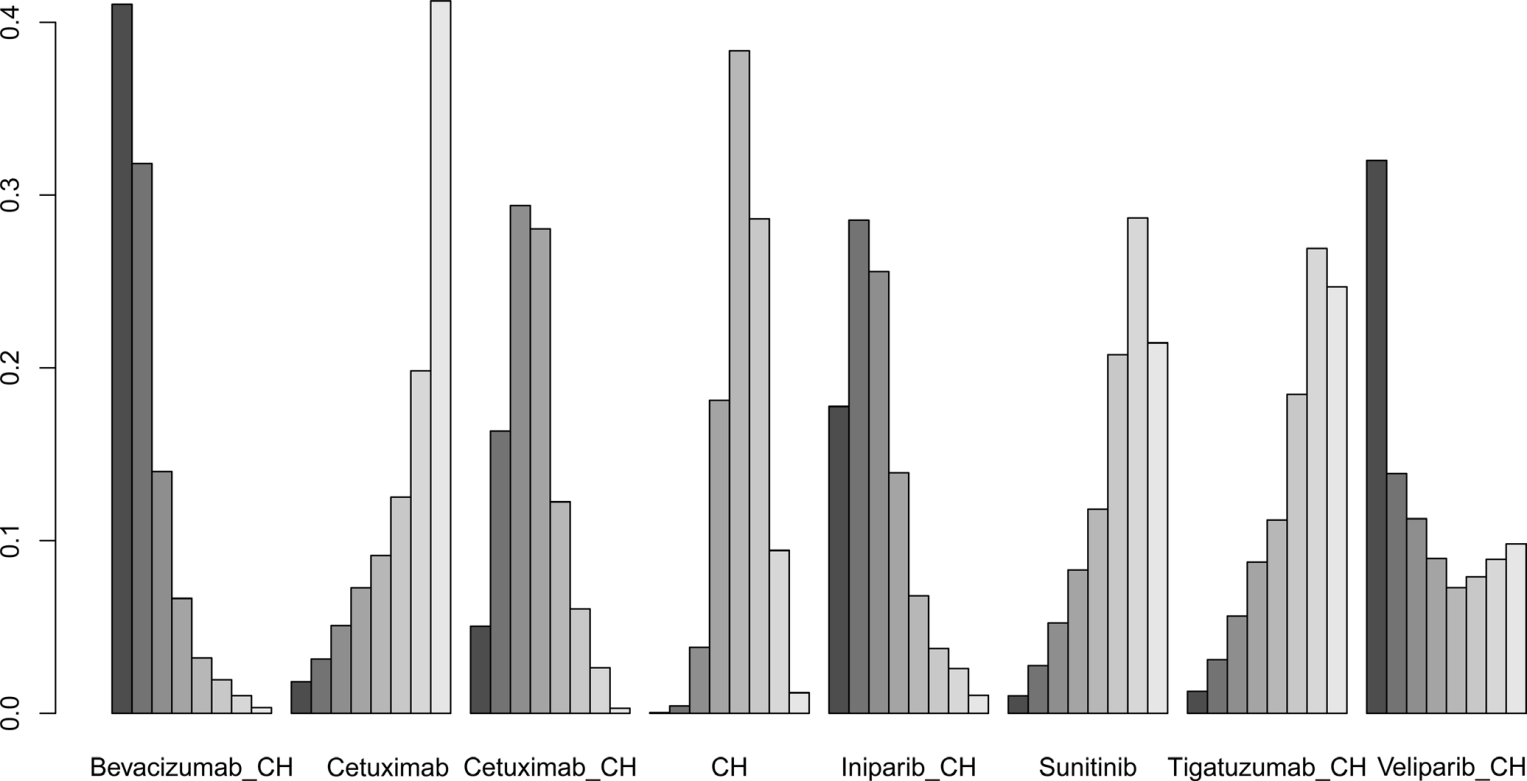
Results of treatment rank for ORR

Eight studies [16, 17, 19–22, 29, 30] (956 patients), involving eight treatment arms, reported on ORR (Figure 3C). There were no statistically significant differences for all comparison groups in the improvement of ORR (Figure 8). The results of treatment rank probabilities indicated that bevacizumab+chemotherapy, veliparib+chemotherapy, and iniparib+chemotherapy were the three best treatments (Figure 9).

### Consistency of Bayesian and Frequentist methods

We also performed a Frequentist network meta-analysis for PFS. Relative HR (RHR) values were calculated to compare the robustness of results between Bayesian and Frequentist methods. The results indicated that there were no inconsistencies among all comparison groups, although the rank was slightly inconsistent. The top four treatments of P-score were still bevacizumab+chemotherapy, veliparib+chemotherapy, sorafenib+chemotherapy, and cetuximab (Supplementary Table 3).

### Publications bias

The comparison-adjusted funnel plot can be found in Supplementary Figures 1–3. Different colors correspond to different comparisons. The results showed that the probability of bias of small-study effects was small for three outcomes.

## DISCUSSION

The treatment of TNBC remains a major clinical challenge due to uncommonness, aggressiveness, and impressive heterogeneity [31]. Cytotoxic chemotherapy remains the standard treatment. Our network meta-analysis collected the currently available RCTs to assess the survival outcomes and ORR of targeted agents combined with chemotherapy in the treatment of advanced/metastatic TNBC. The results of direct comparisons showed that only bevacizumab plus chemotherapy had a significant improvement in PFS when compared with chemotherapy alone. However, there were no significant differences in improvement of PFS, OS, and ORR for other direct comparison groups and all indirect comparison groups. Clinical decisions about the choice of treatments can be recommended based on the probability results of ranking when the differences in effect size of different treatments are small [32]. The rankings of targeted agents plus chemotherapy were made, although the statistical differences were not found in our indirect comparisons. According to the results of treatment rank probabilities, veliparib+chemotherapy, sorafenib+chemotherapy, cetuxi- mab, and bevacizumab+chemotherapy had the largest probabilities to be best in the improvement of PFS. However, the number of included studies and sample sizes were small. The statistical power was insufficient for veliparib+chemotherapy, sorafenib+chemotherapy, and cetuximab, indicating that more studies of targeted agents for advanced/metastatic TNBC are needed. For OS, iniparib+chemotherapy, bevacizumab+chemotherapy, and cetuximab were the top three treatment regimens. Bevacizumab+chemotherapy, veliparib+chemotherapy, and iniparib+chemotherapy were the top three treatment regimens for ORR. Overall, PFS, OS, and ORR favored bevacizumab+chemotherapy, veliparib+chemotherapy, cetuximab, and iniparib+chemotherapy. However, the statistical power was insufficient due to the limited sample sizes.

Previous meta-analysis demonstrated that the pCR rate has achieved a significant improvement in TNBC patients treated with a carboplatin-containing or bevacizumab-containing regimen [33]. However, there were no RCTs to compare the clinical efficacy of bevacizumab+carboplatin and carboplatin alone, although our study confirmed that PFS favored bevacizumab+chemotherapy. While the differences in effect size were not statistically significant for veliparib+chemotherapy, it had a high rank probability to be the best treatment option. Kummar et al.’ study indicated that the addition of veliparib to cyclophosphamide did not improve the response rate for TNBC. Careful consideration will be needed for future trial designs involving veliparib [30]. Only two studies were included to compared iniparib+chemotherapy to chemotherapy alone with favored treatment rank probabilities. The results of phase 2 trials showed that the addition of iniparib to chemotherapy improved the clinical benefit and survival of patients with metastatic TNBC without significantly increasing toxic effects [17]. The phase 3 trial of iniparib indicated that no statistically significant difference was observed for OS (HR = 0.88; 95% CI, 0.69 to 1.12; *P* = 0.28) or PFS (HR = 0.79; 95% confidence interval [CI], 0.65 to 0.98; *P* = 0.027) [28].

No single-targeted agents have been approved for TNBC, and combining two or more targeted agents might be considered as a more rational and optimal approach to treat TNBC [31]. Our network meta-analysis showed that bevacizumab+chemotherapy had better efficacy in the improvement of PFS than chemotherapy alone, although statistical differences were not found for other comparison groups. However, clinicians should be cautious when considering the implications of targeted agents in the patients of TNBC because the statistical power was insufficient, and the safety of targeted agents was not a concern in the present study.

The methodological quality was moderate to high for included studies. All included RCTs were from multicenter studies, indicating that our original data was more reliable than that of single-center RCTs [34]. The sample sizes of included RCTs ranged from 39 to 519. Small to moderately sized trials have stronger effect estimates than larger trials [35]. Therefore, we used a comparison-adjusted funnel plot to identify the bias of small-study effects. The results showed that the probability of bias of small-study effects was low. Although eight open-label studies were included, performance bias and measurement bias remained small because all outcomes of interest were objective.

There were some limitations in our study. First, the number of studies included was relatively small, and the differences of size ranges were large. In particular, only one RCT was identified for chemotherapy combined with lapatinib, sorafenib, sunitinib, tigatuzumab, and veliparib, respectively. More blinded, rigorously designed RCTs are needed. Second, there were no head-to-head RCTs to compare the efficacy between different targeted agents combined with chemotherapy, and thus evaluation of inconsistency was impossible. Third, we did not detail the composition of chemotherapy because different compositions of chemotherapy were found in the limited number of studies included. Fourth, we only focused on advanced/metastatic TNBC. More evidence-based data was needed to confirm the clinical efficacy of targeted agents for early TNBC. Finally, limited outcomes were reported in each study included, preventing the performance of comparisons between some targeted agents. However, our study also had some strengths. This is the first network meta-analysis of RCTs to systematically compare the PFS, OS, and ORR of targeted agents and chemotherapy in advanced/metastatic TNBC.

In summary, this network meta-analysis showed that only bevacizumab+chemotherapy resulted in a significant improvement in PFS when compared with chemotherapy alone for advanced/metastatic TNBC. However, there were no significant differences between different combination regimens of targeted agents’ in the improvement of PFS, OS, and ORR. Further investigation of novel therapies for TNBC is required to improve patient outcomes.

## MATERIALS AND METHODS

### Registration information

The reporting of this network meta-analysis adhered to the Preferred Reporting Items for *Systematic Reviews* and Meta-analyses (PRISMA) extension statement for the reporting of systematic reviews incorporating network meta-analyses of health care interventions [36]. The network meta-analysis was registered in the international prospective register of systematic reviews (PROSPERO) and the registration number is CRD42014014299.

### Search strategy

A systematic search was performed using PubMed, EMBASE, the Cochrane Central Register of Controlled Trials (CENTRAL), Web of Science (via ISI Web of Knowledge), BIOSIS Previews (via ISI Web of Knowledge), and Chemical Abstracts (CA). The search terms were as follows: triple-negative breast cancer*, breast tumor*, breast carcinoma*, breast neoplasm*, random*, randomized controlled trial*, randomized trial*. The search strategy was developed by Ge L and Tian JH (more than 10 years’ experience as information specialist). Full details of the search strategy regarding PubMed and EMBASE are included in Supplementary Text 1. There were no language restrictions on our search. The last search was updated November 18, 2016. The references of included articles and reviews were tracked to identify other relevant studies.

### Inclusion criteria

All RCTs that met following eligibility criteria were included: Types of participants: female sex, age of 18 years or older, and advanced/metastatic TNBC patients who had been histologically documented as ER-negative, PR-negative, and not having overexpression of HER-2‚ Types of interventions: targeted agents combined with chemotherapy. ƒ Type of studies: RCTs that compared different targeted agents to chemotherapy for treating TNBC. We excluded non-randomized, phase I clinical trials and studies that compared the efficacy of TNBC to non-TNBC. „ Types of outcome measures: PFS (defined as the time from randomization to confirmation of disease progression or death), OS (defined as the time from randomization until the date of death), and ORR (defined as the percentage of patients who had a complete response, a partial response, or stable disease for at least 6 months).

### Study selection

Two independent reviewers examined the title and abstract of studies found in the search to identify relevant studies according to inclusion criteria. Then, full-text versions of all potentially relevant studies were obtained. Full-texts were examined independently by pairs of reviewers. Excluded trials and the reason for their exclusion were listed and examined by a third reviewer.

### Data extraction and assessment of risk of bias

A standard data abstraction form was created using Microsoft Excel 2013 (Microsoft Corp, Redmond, WA, www.microsoft.com) to collect data of interest. Two independent reviewers extracted data, including the author, year of publication, study arms, sample, median age, journals, median OS, median PFS, and outcomes, and conflict was resolved by discussion.

The risk of bias was evaluated according to the Cochrane Handbook version 5.1.0 [37], including the method of random sequence generation, allocation concealment, blinding, incomplete outcome data, selective reporting, and other bias. Disagreements were resolved by a third reviewer.

### Geometry of the network

Network plots were drawn to describe and present the geometry of different targeted agents using R-3.3.2 software (R Foundation for Statistical Computing, Vienna, Austria). Nodes were used to represent different interventions and edges to represent the head-to-head comparisons between interventions.

### Statistical methods

A Bayesian network meta-analysis was performed using the ‘gemtc’ *version 0.8.1* package of R-3.3.2 software [38]. The function mtc.run was used to generate samples using the Markov chain Monte Carlo sampler. Four Markov chains were run simultaneously. We set 5000 simulations for each chain as the ‘burn-in’ period. Then, posterior summaries were based on 50,000 subsequent simulations. The model convergence was assessed using Brooks-Gelman-Rubin plots [39].

HR with 95% CrIs were used for PFS and OS. Odds ratios (OR) with 95% CrIs were used for ORR. Rank probabilities indicate the probability for each treatment to be best, second best, etc. Clinical decisions about the choice of treatments can be recommended based on the probability results of ranking when the differences in effect size of different treatments are small [32]. The ‘gemtc’ package provides a matrix of the treatment rank probabilities, as well as a plot of the rank probabilities.

Clinical and methodological heterogeneity were assessed by carefully examining the characteristics and design of included trials. Heterogeneity of treatment effects across head-to-head trials was assessed by I^2^ statistics using the mtc.anohe command of the ‘gemtc’ package. If the I^2^ was ≤ 50%, it suggested that there was no statistical heterogeneity, and the fixed effects model was used for meta-analysis. If the I^2^ was > 50%, we explored sources of heterogeneity by subgroup analysis and meta-regression using effect modifiers. If there was no clinical heterogeneity, the random effects model was used to perform meta-analysis.

If a loop connecting three arms existed, inconsistency between direct and indirect comparisons was evaluated by the node-splitting method [40]. A comparison-adjusted funnel plot was used to identify whether there the small sample effect existed between intervention networks.

A frequentist network meta-analysis was also conducted for PFS using the ‘netmeta’ version 0.9–2 package of R-3.3.2 software [41]. RHR was calculated to assess the consistency of results of Bayesian and Frequentist methods.

## SUPPLEMENTARY MATERIALS FIGURES AND TABLES






