# Supplementary material for: A network meta-analysis on the efficacy of targeted agents in combination with chemotherapy for treatment of advanced/metastatic triple-negative breast cancer
Source: Oncotarget. 2017 Jul 8;8(35):59539–51. doi: 10.18632/oncotarget.19102 (PMC5601753; doi:10.18632/oncotarget.19102)
Supplement: Supplementary file 1 [file oncotarget-08-59539-s001.pdf]

A network meta-analysis on the efficacy of targeted agents in combination with chemotherapy for treatment of advanced/metastatic triple-negative breast cancer

SUPPLEMENTARY MATERIALS

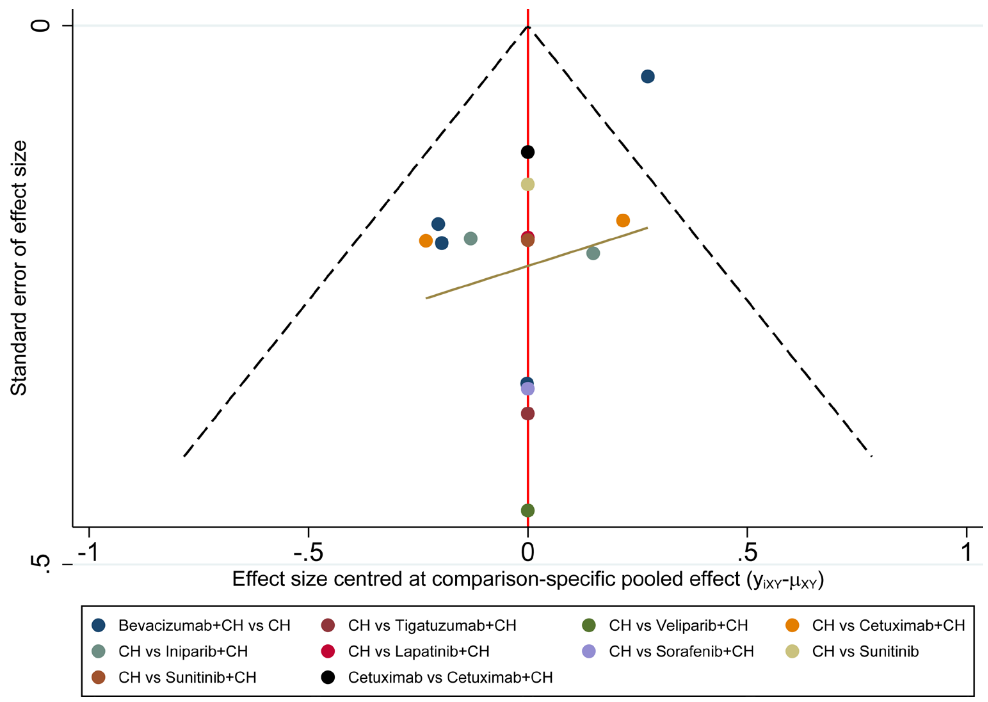

Supplementary Figure 1: Comparison-adjusted funnel plot for PFS.

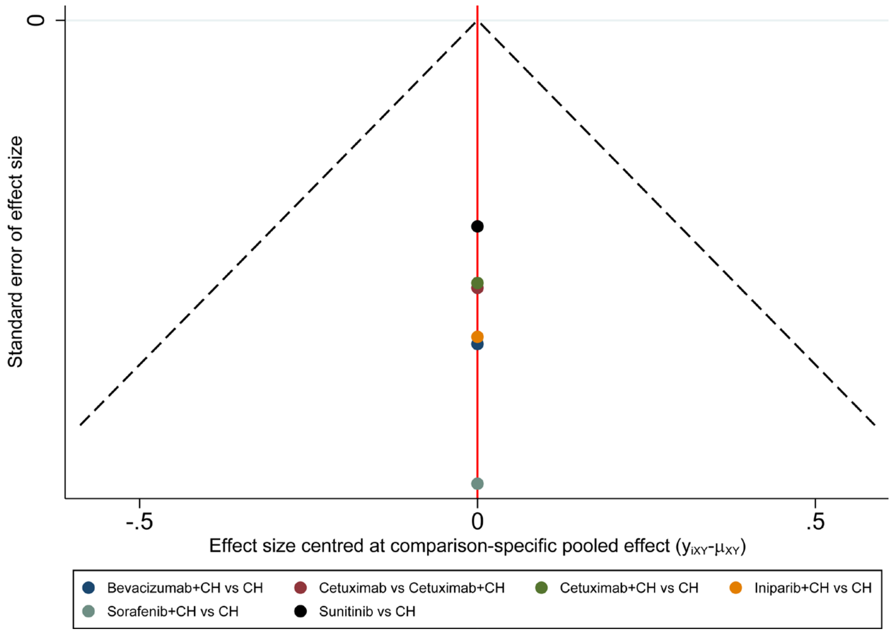

Supplementary Figure 2: Comparison-adjusted funnel plot for OS.

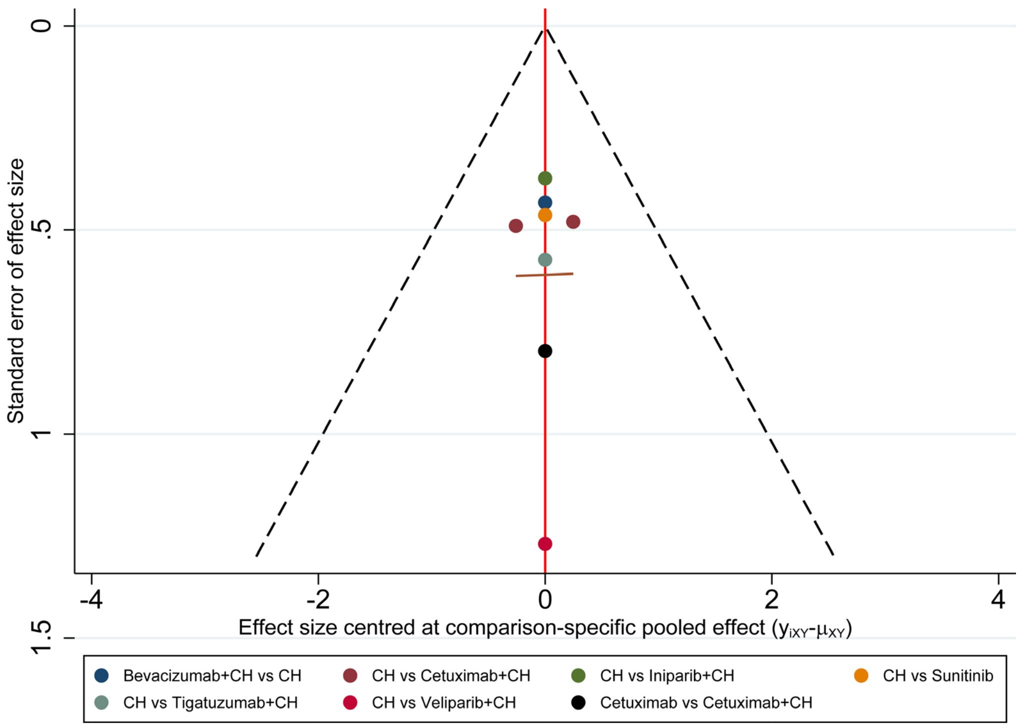

Supplementary Figure 3: Comparison-adjusted funnel plot for ORR.

Supplementary Table 1: The details of targeted agents and chemotherapy regimens. See Supplementary_Table_1

Supplementary Table 2: The details of risk of bias for each study

Studies	Adequate sequence generation	Adequate allocation concealment	Blinding of participants and personnel	Blinding of outcome assessment	Incomplete outcome data addressed	Free of selective reporting	Other bias
RIBBON-2	Low	Low	Low	Unclear	Low	Low	High
NCT00528567	Low	Low	Open-label	Unclear	Low	Low	High
EGF30001	Low	Low	Double-blind	High	Low	Low	High
NCT00246571	Unclear	Unclear	Open-label	Unclear	Low	Low	Unclear
NCT00633464	Low	Low	Open-label	Unclear	Low	Low	Low
TBCRC 001	Unclear	Unclear	Low	Unclear	Low	Low	Low
NCT00463788	Low	Low	Open-label	Unclear	Low	Low	Low
SOLTI0701	Low	Low	Double-blind	Unclear	Low	Low	High
Bergh J 2012	Unclear	Low	Open-label	Unclear	Low	Low	High
AVADO	Low	Low	Double-blind	High	Low	Low	High
E2100	Low	Low	Open-label	Unclear	Low	Low	High
RIBBON-1	Low	Low	Low	Unclear	Low	Low	High
O'Shaughnessy J 2014	Low	Low	open-label	Low	Low	Low	Low
TBCRC 019	Unclear	Unclear	Unclear	Unclear	Low	Low	Low
NCT01306032	Unclear	Unclear	Open-label	High	Low	Low	Low

Supplementary Table 3: The results of consistency of Bayesian and Frequentist methods for PFS. See Supplementary_Table_3

SUPPLEMENTARY TEXT 1: THE SEARCH STRATEGY

The search strategy of PubMed:

#1 “Triple Negative Breast Neoplasms”[Mesh]

#2 “breast Cancer*” OR “breast tumor*” OR “breast carcinoma*” OR “breast neoplasm”[Title/Abstract]

#3 “Breast Neoplasms”[Mesh]

#4 #2 OR #3

#5 “triple negative”[Title/Abstract]

#6 Random* OR randomized controlled trial* OR randomized trial* OR Randomized Controlled Trial[ptyp] OR “Randomized Controlled Trials as Topic”[Mesh]

#7 #1 OR #4

#8 #5 AND #7 AND #6

The search strategy of EMBASE:

#1 ‘breast cancer’/exp OR ‘breast cancer’ OR ‘breast tumor’/exp OR ‘breast tumor’ OR ‘breast carcinoma’/exp OR ‘breast carcinoma’ OR ‘breast neoplasm’

#2 ‘triple negative’/exp OR ‘triple negative’

#3 #1 AND #2 AND [randomized controlled trial]/lim
