# Supplementary material for: A network meta-analysis on the efficacy of targeted agents in combination with chemotherapy for treatment of advanced/metastatic triple-negative breast cancer
Source: Oncotarget. 2017 Jul 8;8(35):59539–51. doi: 10.18632/oncotarget.19102 (PMC5601753; doi:10.18632/oncotarget.19102)
Supplement: Supplementary file 2 [file oncotarget-08-59539-s002.doc]

**A network meta-analysis on the efficacy of targeted agents in combination with chemotherapy for treatment of advanced/metastatic triple-negative breast cancer**

**Supplementary Table 1: The details of targeted agents and chemotherapy regimens**

| **Study** | **Arm** | **Sample** |
| --- | --- | --- |
| Brufsky A 2011 | bevacizumab+chemotherapy | Bevacizumab 10 mg/kg every 2 weeks or 15 mg/kg every 3 weeks, Taxane (paclitaxel 90 mg/m2 d1, 8, 15 q4w or paclitaxel 175 mg/m 2, nab-paclitaxel 260 mg/m2 or docetaxel 75-100 mg/m2 q3w), Gemcitabine (1,250 mg/m2 d1, 8 q3w), Capecitabine (1,000 mg/m2 bid d1-14 q3w), Vinorelbine (30 mg/m2 d1, 8, 15 q3w) |
|  | chemotherapy | Placebo, Taxane (paclitaxel 90 mg/m2 d1, 8, 15 q4w or paclitaxel 175 mg/m 2, nab-paclitaxel 260 mg/m2 or docetaxel 75-100 mg/m2 q3w), Gemcitabine (1,250 mg/m2 d1, 8 q3w), Capecitabine (1,000 mg/m2 bid d1-14 q3w), Vinorelbine (30 mg/m2 d1, 8, 15 q3w) |
| O’Shaughnessy J 2011 | iniparib+chemotherapy | Control group regimen combined with IV iniparib (4.0 mg per kilogram) over a 60-minute period, on days 1, 4, 8, and 11 |
|  | chemotherapy | During each 21-day period, on days 1 and 8, intravenous gemcitabine (1000 mg per square meter of bodysurface area) over a 30-minute period and carboplatin (at a dose equivalent to an area under the concentration–time curve of 2) over a 60-minute period. |
| Finn RS 2009 | lapatinib+chemotherapy | Paclitaxel 175 mg/m^2^ IV every 3 weeks with either oral lapatinib 1,500 mg daily or placebo |
|  | chemotherapy | Paclitaxel 175 mg/m^2^ IV every 3 weeks with placebo |
| Curigliano G 2013 | sunitinib | Sunitinib 37.5 mg starting dose/3 weeks, dose reduction to 25 mg/day was also permitted for recurring toxicity |
|  | chemotherapy | Capecitabine Oral 2000-2500 mg/m^2^, gemcitabine IV 800-1250 mg/m^2^, vinorelbine IV 25-30 mg/m^2^, docetaxel IV 75-100 mg/m^2^, paclitaxel IV 175-200 mg/m^2^ |
| Trédan O 2014 | cetuximab+chemotherapy | Ixabepilone 40 mg/m2 as a 3-hour intravenous (I.V.) infusion on day 1 every 21 days |
|  | chemotherapy | Ixabepilone 40 mg/m2 as a 3-hour intravenous (I.V.) infusion on day 1 every 21 days with cetuximab 400 mg/m^2^ |
| Carey LA 2012 | cetuximab | Cetuximab 400 mg/m^2^ IV |
|  | cetuximab+chemotherapy | Cetuximab 400 mg/m^2^ IV, carboplatin was administered at an AUC of 2 IV on days 1, 8, and 15 of each 28-day cycle |
| Baselga J 2013 | cetuximab+chemotherapy | Cetuximab 400 mg/m^2^ IV, cisplatin 75 mg/m^2^ on day 1, every 3 weeks, for six cycles |
|  | chemotherapy | Cisplatin 75 mg/m^2^ on day 1, every 3 weeks, for six cycles |
| Baselga J 2012 | sorafenib+chemotherapy | Capecitabine 1,000 mg/m^2^ orally twice a day for days 1 to 14 of every 21-day cycle with sorafenib 400 mg orally twice a day |
|  | chemotherapy | Capecitabine 1,000 mg/m^2^ orally twice a day for days 1 to 14 of every 21-day cycle with placebo |
| Bergh J 2012 | sunitinib+chemotherapy | Sunitinib 37.5 mg/d, days 2 to 15 every 3 weeks; and docetaxel 75 mg/m^2^, day 1 every 3 weeks |
|  | chemotherapy | Docetaxel 100 mg/m^2^ every 3 weeks |
| Pivot X 2011 | bevacizumab+chemotherapy | Bevacizumab7.5 mg/kg or bevacizumab 15 mg/kg, 9 cycles +docetaxel (100 mg/m^2^) 3-weekly |
|  | chemotherapy | Docetaxel (100 mg/m^2^) 3-weekly |
| Miller K 2007 | bevacizumab+chemotherapy | Paclitaxel per square meter of body-surface area on days 1, 8, and 15 every 4 weeks with 10 mg of bevacizumab per kilogram of body weight on days 1 and 15 |
|  | chemotherapy | Paclitaxel per square meter of body-surface area on days 1, 8, and 15 every 4 weeks |
| Robert NJ 2011 | bevacizumab+chemotherapy | Bevacizumab (15 mg/kg every 3 weeks)+capecitabine (2,000 mg/m^2^ for 14 days) |
|  | chemotherapy | capecitabine (2,000 mg/m^2^ for 14 days) |
|  | bevacizumab+chemotherapy | bevacizumab (15 mg/kg every 3 weeks)+taxane/anthracycline |
|  | chemotherapy | Taxane (nab-paclitaxel 260 mg/m2, docetaxel 75 or 100 mg/m^2^)/anthracycline |
| O’Shaughnessy J 2014 | iniparib+chemotherapy | Gemcitabine 1,000 mg/m^2^ and carboplatin area under the curve= 2 intravenously on days 1 and 8 with or without iniparib 5.6 mg/kg intravenously on days 1, 4, 8, and 11 of each 21-day cycle |
|  | chemotherapy | Gemcitabine 1,000 mg/m^2^ and carboplatin area under the curve= 2 intravenously on days 1 and 8 |
| Forero-Torres A 2015 | tigatuzumab+chemotherapy | IV albumin-bound paclitaxel on days 1, 8, and 15 (100 mg/m^2^) at 28-days interval with tigatuzumab intravenously on days 1 and 15 of every cycle (10 mg/kg loading dose followed by 5 mg/kg every other week) |
|  | chemotherapy | IV albumin-bound paclitaxel on days 1, 8, and 15 (100 mg/m^2^) at 28-days interval |
| Kummar S 2016 | veliparib+chemotherapy | Oral veliparib 60 mg once daily throughout a 21-day cycle, cyclophosphamide 50 mg once daily |
|  | chemotherapy | Cyclophosphamide 50 mg once daily |
