# Supplementary material for: A network meta-analysis on the efficacy of targeted agents in combination with chemotherapy for treatment of advanced/metastatic triple-negative breast cancer
Source: Oncotarget. 2017 Jul 8;8(35):59539–51. doi: 10.18632/oncotarget.19102 (PMC5601753; doi:10.18632/oncotarget.19102)
Supplement: Supplementary file 3 [file oncotarget-08-59539-s003.doc]

**A network meta-analysis on the efficacy of targeted agents in combination with chemotherapy for treatment of advanced/metastatic triple-negative breast cancer**

**Supplementary Table 3:** The results of consistency of Bayesian and Frequentist methods for PFS

| **Comparisons** | **Estimates, HR (95%CI)** | | **RHR (95%CI)** | **Rank** | |
| --- | --- | --- | --- | --- | --- |
|  | **Bayesian** | **Frequentist** |  | **Bayesian** | **Frequentist** |
| **CH (Reference)** |  |  |  |  |  |
| Bevacizumab+CH | 0.62 (0.41,0.87) | 0.60 (0.45,0.82) | 1.03 (0.64,1.67) | 4 | **1** |
| Iniparib+CH | 0.68 (0.40, 1.2) | 0.68 (0.44,1.06) | 1.0 (0.49,2.02) | 5 | 5 |
| Lapatinib+CH | 1.3 (0.59,2.7) | 1.25 (0.68,2.31) | 1.04 (0.39,2.76) | 9 | 10 |
| Sunitinib | 1.2 (0.56,2.4) | 1.16 (0.66,2.03) | 1.03 (0.41,2.59) | 8 | 9 |
| Cetuximab+CH | 0.83 (0.49,1.4) | 0.83 (0.54,1.28) | 1.0 (0.51,1.97) | 10 | 6 |
| Cetuximab | 0.61 (0.25,1.5) | 0.61 (0.31,1.20) | 1.0 (0.33,3.07) | **3** | **3** |
| Sorafenib+CH | 0.60 (0.24,1.5) | 0.60 (0.27,1.36) | 0.98 (0.31,3.07) | **2** | 4 |
| Sunitinib+CH | 0.96 (0.45,2.0) | 0.96 (0.52,1.78) | 1.0 (0.38,2.63) | 7 | 7 |
| Tigatuzumab+CH | 1.0 (0.40, 2.7) | 1.04 (0.44,1.06) | 0.96 (0.34, 2.75) | 6 | 8 |
| Veliparib+CH | 0.55 (0.19, 1.6) | 0.55 (0.20,1.50) | 1.0 (0.23,4.33) | **1** | **2** |
| **Bevacizumab+CH (Reference)** | |  |  |  |  |
| Iniparib+CH | 1.1 (0.59,2.2) | 1.13 (0.66,1.92) | 0.97 (0.42,2.27) |  |  |
| Lapatinib+CH | 2.0 (0.90,4.9) | 2.07 (1.04,4.10) | 0.97 (0.32,2.87) |  |  |
| Sunitinib | 1.9 (0.87,4.4) | 1.92 (1.02,3.62) | 0.99 (0.35,2.77) |  |  |
| Cetuximab+CH | 1.3 (0.73,2.7) | 1.38 (0.81,2.32) | 0.94 (0.41,2.18) |  |  |
| Cetuximab | 0.99 (0.39,2.7) | 1.00 (0.48,2.12) | 0.99 (0.29,3.35) |  |  |
| Sorafenib+CH | 0.98 (0.37,2.7) | 0.99 (0.42,2.37) | 0.99 (0.27,3.70) |  |  |
| Sunitinib+CH | 1.6 (0.69,3.8) | 1.59 (0.80,3.16) | 1.01 (0.34,3.01) |  |  |
| Tigatuzumab+CH | 1.7 (0.63,4.9) | 1.72 (0.70,4.25) | 0.99 (0.25,3.87) |  |  |
| Veliparib+CH | 0.90 (0.29,2.9) | 0.91 (0.32,2.59) | 0.99 (0.21,4.68) |  |  |
| **Iniparib+CH (Reference)** | |  |  |  |  |
| Lapatinib+CH | 1.8 (0.72,4.7) | 1.84 (0.86,3.92) | 0.99 (0.29,3.27) |  |  |
| Sunitinib | 1.7 (0.69,4.2) | 1.70 (0.83,3.48) | 1.0 (0.32,3.17) |  |  |
| Cetuximab+CH | 1.2 (0.57,2.6) | 1.22 (0.66,2.26) | 0.98 (0.37,2.61) |  |  |
| Cetuximab | 0.89 (0.32,2.5) | 0.89 (0.40,2.01) | 1.0 (0.27,3.69) |  |  |
| Sorafenib+CH | 0.88 (0.30,2.6) | 0.88 (0.35,2.23) | 1.0 (0.24,4.15) |  |  |
| Sunitinib+CH | 1.4 (0.56,3.6) | 1.41 (0.66,3.01) | 0.99 (0.30,3.30) |  |  |
| Tigatuzumab+CH | 1.5 (0.51,4.6) | 1.53 (0.58,3.99) | 0.98 (0.23,4.23) |  |  |
| Veliparib+CH | 0.81 (0.24,2.7) | 0.81 (0.27,2.41) | 1.0 (0.20,5.11) |  |  |
| **Lapatinib+CH (Reference)** | |  |  |  |  |
| Sunitinib | 0.93 (0.32,2.6) | 0.93 (0.40,2.13) | 1.0 (0.26,3.82) |  |  |
| Cetuximab+CH | 0.66 (0.26,1.7) | 0.67 (0.31,1.41) | 0.99 (0.29,3.29) |  |  |
| Cetuximab | 0.49 (0.15,1.6) | 0.49 (0.19,1.22) | 1.0 (0.22,4.50) |  |  |
| Sorafenib+CH | 0.48 (0.16,1.6) | 0.48 (0.17,1.33) | 1.0 (0.21,4.68) |  |  |
| Sunitinib+CH | 0.77 (0.26,2.2) | 0.77 (0.32,1.84) | 1.0 (0.25,3.98) |  |  |
| Tigatuzumab+CH | 0.83 (0.25,2.8) | 0.83 (0.29,2.38) | 1.0 (0.20,4.96) |  |  |
| Veliparib+CH | 0.44 (0.12,1.6) | 0.44 (0.14,1.43) | 1.0 (0.18,5.70) |  |  |
| **Sunitinib (Reference)** | |  |  |  |  |
| Cetuximab+CH | 0.72 (0.30,1.8) | 0.72 (0.35,1.45) | 1.0 (0.32,3.14) |  |  |
| Cetuximab | 0.52 (0.17,1.7) | 0.52 (0.22,1.27) | 1.0 (0.24,4.25) |  |  |
| Sorafenib+CH | 0.52 (0.16,1.7） | 0.52 (0.19,1.39) | 1.0 (0.21,4.69) |  |  |
| Sunitinib+CH | 0.83 (0.29,2.4) | 0.83 (0.36,1.91) | 1.0 (0.26,3.84) |  |  |
| Tigatuzumab+CH | 0.90 (0.27,2.9) | 0.90 (0.32,2.49) | 1.0 (0.21,4.80) |  |  |
| Veliparib+CH | 0.48 (0.13,1.7) | 0.47 (0.15,1.49) | 1.02 (0.18,5.72) |  |  |
| **Cetuximab+CH (Reference)** | |  |  |  |  |
| Cetuximab | 0.73 (0.36,1.5) | 0.73 (0.43,1.24) | 1.0 (0.41,2.43) |  |  |
| Sorafenib+CH | 0.72 (0.25,2.1) | 0.72 (0.29,1.81) | 1.0 (0.25,4.07) |  |  |
| Sunitinib+CH | 1.2 (0.45,2.9) | 1.15 (0.54,2.45) | 1.04 (0.31,3.46) |  |  |
| Tigatuzumab+CH | 1.3 (0.42,3.7) | 1.25 (0.48,3.25) | 1.04 (0.24,4.43) |  |  |
| Veliparib+CH | 0.67 (0.20,2.2) | 0.66 (0.22,1.97) | 1.02 (0.20,5.15) |  |  |
| **Cetuximab (Reference)** | |  |  |  |  |
| Sorafenib+CH | 0.99 (0.28,3.5) | 0.99 (0.34,2.87) | 1.0 (0.19,5.22) |  |  |
| Sunitinib+CH | 1.6 (0.49,5.0) | 1.58 (0.63,3.97) | 1.01 (0.23,4.46) |  |  |
| Tigatuzumab+CH | 1.7 (0.47,6.2) | 1.71 (0.57,5.12) | 0.99 (0.18,5.41) |  |  |
| Veliparib+CH | 0.91 (0.23,3.6) | 0.90 (0.27,3.05) | 1.01 (0.16,6.32) |  |  |
| **Sorafenib+CH (Reference)** | |  |  |  |  |
| Sunitinib+CH | 1.6 (0.49,5.3) | 1.60 (0.57,4.45) | 1.0 (0.21,4.82) |  |  |
| Tigatuzumab+CH | 1.7 (0.46.6.5) | 1.73 (0.53,5.65) | 0.98 (0.16,5.80) |  |  |
| Veliparib+CH | 0.92 (0.22,3.8) | 0.91 (0.25,3.34) | 1.01 (0.15,6.94) |  |  |
| **Sunitinib+CH (Reference)** | |  |  |  |  |
| Tigatuzumab+CH | 1.1 (0.32,3.7) | 1.08 (0.38,3.11) | 1.01 (0.20,5.11) |  |  |
| Veliparib+CH | 0.58 (0.15,2.1) | 0.57 (0.18,1.86) | 1.02 (0.17,5.93) |  |  |
| **Tigatuzumab+CH (Reference)** | |  |  |  |  |
| Veliparib+CH | 0.53 (0.13,2.2) | 0.53 (0.14,1.97) | 1.0 (0.14,6.93) |  |  |
